# Paediatric rotations in undergraduate medical education in Switzerland: Meeting students’ expectations and the goals of the competency-based learning catalogue PROFILES

**DOI:** 10.3205/zma001718

**Published:** 2024-11-15

**Authors:** Lya Baumann, Beatrice Latal, Michelle Seiler, Sabine Kroiss Benninger

**Affiliations:** 1 University of Zurich, Zurich, Switzerland; 2 University Children’s Hospital Zurich, Eleonorenstiftung, Zurich, Switzerland

**Keywords:** undergraduate medical education, Entrustable Professional Activities, paediatric clerkship, teaching in clinical setting, competency-based training

## Abstract

**Introduction:**

The competency-based catalogue of learning objectives “Principal Relevant Objectives and Framework for Integrative Learning and Education in Switzerland” (PROFILES) based on Entrustable Professional Activities (EPAs) was newly introduced in 2018 in undergraduate medical education in Switzerland. Clerkships provide opportunities for students to train clinical skills and competencies within the curriculum. This study aims to assess the students’ experiences during paediatric clerkships and whether they achieve the expected competency level of certain EPAs by the end of their training.

**Methods:**

An online survey was conducted among all 316 students in their last year of medical school (3^rd^ year master) enrolled at the University of Zurich. A total of 113 students who had completed a clerkship in paediatrics in different hospitals, were asked about their general expectations and experiences, and to rate their achievement of competency levels in 26 selected EPAs. An EPA was considered accomplished if a minimum of 2/3 of all students reached at least level 3.

**Results:**

Paediatric clerkship was generally viewed as positive experience by most students. However, a desire for more integration into clinical teams, increased training in clinical skills, and feedback was expressed. The expected level 3 of competency (indirect supervision) was achieved in 14 out of 26 EPAs by at least 2/3 of students. Level 3 was however not reached for more specific EPAs such as neonatal examination, rating of psychomotor and pubertal development, and clinical reasoning.

**Conclusion:**

Paediatric clerkships are regarded as valuable clinical training opportunities. To enhance the learning of competencies, integration into clinical teams and faculty training is crucial. The implementation of EPAs in the clinical context aligns with these goals.

## Zusammenfassung

**Ziel:**

Der Kompetenzbasierte Lernzielkatalog „Principal Relevant Objectives and Framework for Integrative Learning and Education in Switzerland“ (PROFILES) beruhend auf sogenannten Entrustable Professional Activities (EPAs=anvertraute professionelle Aktivitäten) wurde 2018 in der Schweiz neu in die medizinische Ausbildung eingeführt. Klinische Rotationen innerhalb des Curriculums bieten den Studierenden die Gelegenheit klinische Fertigkeiten und Kompetenzen zu trainieren. Diese Studie hat zum Ziel herauszufinden, welche Erfahrungen der Studierenden während einer Rotation in der Pädiatrie machen und ob sie die erwarteten Kompetenz-Niveaus bestimmter EPAs am Ende Ihrer Ausbildung erreichen.

**Methoden:**

Es wurde eine elektronische Umfrage unter allen 316 Studierenden ihres letzten Jahres des Medizinstudiums (3. Jahr Masterstudium) der Universität Zürich durchgeführt. Insgesamt wurden 113 Studierende, die eine pädiatrische Rotation in verschiedenen Krankenhäusern abgeschlossen hatten, zu ihren allgemeinen Erwartungen und Erfahrungen befragt, und gebeten Ihr Abschneiden hinsichtlich Kompetenzniveau in 26 ausgewählten EPAs einzuschätzen. Eine EPA wurde als erfüllt beurteilt, wenn mindestens 2/3 aller Studierenden mindestens Level 3 erreicht hatten.

**Results:**

Die pädiatrische Rotation wurde von den meisten Studierenden insgesamt als positive Erfahrung betrachtet. Jedoch wurde der Wunsch nach mehr Integration in die klinischen Teams, mehr Training von klinischen Fertigkeiten und mehr Feedback ausgedrückt. Das erwartete Kompetenzlevel 3 (indirekte Supervision) wurde für 14 von 26 EPAs von mindestens 2/3 der Studierenden erreicht. Level 3 wurde jedoch nicht für spezifischere EPAs erreicht, wie Neugeborenenuntersuchung, Einschätzung der psychomotorischen und Pubertäts-Entwicklung, und klinisches Denken.

**Schlussfolgerung:**

Pädiatrie-Rotationen werden als wertvolle klinische Lern- und Übungs-Gelegenheiten eingeschätzt. Um das Lernen von Kompetenzen zu verstärken, ist die Einbindung in das klinische Team und Training des Lehrkörpers entscheidend. Die Implementierung der EPAs im klinischen Kontext entspricht dieser Zielsetzung.

## 1. Introduction

In Switzerland, all medical schools have integrated clerkships in the 2^nd^ or 3^rd^ year of the master program. These clerkships provide unique opportunities to train specific clinical skills and competencies, gain insights into different specialities, and prepare students for everyday clinical practice. The Joint Commission of the Swiss Medical Schools (SMIFK/CIMS) has introduced the newly revised competency-based catalogue of learning objectives *“****P****rincipal ****R****elevant ****O****bjectives and ****F****ramework for ****I****ntegrative ****L****earning and ****E****ducation in ****S****witzerland”* (PROFILES) [1]. Since 2018, this catalogue serves as a framework for the curricula of all national medical schools and was first used as the basis for the federal licencing exam in 2021 [2]. It introduces Entrustable Professional Activities (EPAs) into undergraduate medical education for the first time, specifying 9 core EPAs with a subset of 161 specific tasks as competency-based learning objectives (see table 1 (Tab. 1)).

EPAs represent units of professional practice that can be entrusted to students once they have acquired sufficient competency [3], [4]. Each EPA integrates a variety of competencies and skills and can be assessed at the workplace, allowing for a holistic evaluation of the student’s overall clinical proficiency [5]. A student’s progression is hereby described by the degree of supervision needed to perform a specific EPA, starting at level 1 “only allowed to observe” and proceeding up to level 5 “supervision can be provided to others” (see table 2 (Tab. 2)) [6], [7]. The implementation of EPAs is widely believed to be advantageous, as they focus on a holistic approach assessing clinically relevant competencies, while also offering the opportunity to an improved feedback culture throughout clinical clerkships [4], [8]. However, some challenges regarding the accompanying curricular changes were identified by different medical schools currently implementing EPAs into undergraduate medical education all over the world [4]. Such challenges include the assessment of an effective integration into and/or replacement of parts of curriculum already in place, as well as the success of the implementation being influenced by the willingness and motivation of medical educators to teach and assess EPAs at their workplace [8], [9]. Further, the scope of implementation of EPAs can differ between different teaching hospitals, making an uniform approach regarding curricular changes and as such a consistent standard throughout difficult [8], [9]. Lastly, medical educators need to ensure qualitatively and quantitatively sufficient assessment of EPAs during clerkship to make well-founded entrustment decisions, as well as ensure a way to teach EPAs that are only sporadically encountered during clinical work [8], [9].

The PROFILES catalogue serves as a binding document for Swiss Universities, presuming that students are required to master all 161 EPAs at a minimum level of 3, performing them “under indirect and moderate supervision”. To comply with the new standards set by PROFILES, adjustments to the existing curriculum are required. At the University of Zurich (UZH), clinical courses are an integral part of the curriculum, offering students the opportunity to be introduced to the clinical workplace. From the 3^rd^ year of Bachelor’s until the 3^rd^ and final year of Master’s studies, students train in small groups at various hospitals, acquiring basic clinical skills and knowledge in all specialities. The 2^nd^ year of the medical master program is designated to workplace-based training in the form of clerkships. In this so-called “Wahlstudienjahr” (WSJ), students spend at least 9 months in clerkships of varying duration, ranging from one to three months, at different teaching hospitals. During WSJ, students become part of the clinical team, gaining hands-on experience and improving their skills and competencies under supervision. It also provides valuable insight into different medical fields and specialities, potentially influencing future career plans [10]. Workplace-based assessment in the form of standardized Mini-Clinical Skills Examinations (Mini-CEX) is mandatory throughout WSJ for all medical students in Switzerland, and students maintain a standardized logbook to document their progress. However, until now, these assessments have not been linked to the PROFILES catalogue and EPAs, as there has been no structured implementation of PROFILES into clinical clerkships. As a result, the scope of a student’s experiences during clerkships may vary.

This study aims to investigate the experiences of students enrolled at the UZH during their paediatric clerkships, in different hospitals in and outside Zürich and abroad, and assess the extent to which the currently implemented curriculum meets the requirements of the new PROFILES catalogue. The study seeks to answer questions such as students’ expectations of their paediatric clerkship and how their experiences influence their career choice. Additionally, the study aims to assess the perceived acquisition of clinical skills and competencies during these rotations, as reported by students, and identify areas for improvement, including potential differences among teaching hospitals. This comparison among hospitals was conducted to ascertain whether notable differences exist between them, given the national binding nature of the PROFILES catalogue.

Based on the findings of this study, we will discuss the lessons learned and implications for the current paediatric training in the WSJ and the implementation of the PROFILES catalogue at the University Children’s hospital Zurich. 

## 2. Materials and methods

An online survey was administered to all 6^th^-year medical students enrolled at the University of Zurich, Switzerland (see attachment 1 ). The survey was conducted from 21^st^ of February 2020 to 17^th^ of March 2020, approximately six months after the students had completed their clerkships during WSJ. A questionnaire was developed by a study group comprising the authors, which included three experienced paediatricians and educators, as well as a recent medical graduate. The questionnaire aimed to assess the students’ experiences during their paediatric rotations and their perceived level of competency in selected EPAs at the end of the clerkships.

The first part of the survey included questions where students were asked to rate their overall satisfaction with the paediatric rotation on a 5-point Likert scale (1=not at all; 5=very much). They were further asked if they would recommend the paediatric rotation and the teaching hospital they attended to others, if they intended to pursue a paediatric residency, and if their experience during the clerkship influenced their career decision. Additionally, students were asked to rate their agreement with a series of statements related to their everyday work during the rotation. These statements included subjects such as the opportunity to apply their knowledge and practice skills, teaching and feedback from supervisors, integration into the clinical team, and their opinion on Mini-CEX assessments and the use of a logbook. Responses were rated on a 5-point Likert scale (1=not correct at all; 5=fully correct). Open text comments were allowed.

The second part of the survey involved a self-assessment by the students regarding the level of competency they had reached in selected EPAs. The study group selected 26 EPAs from the PROFILES catalogue that were deemed feasible and relevant for paediatrics. Three of these EPAs were adapted and shortened as they included examinations that were not relevant in the paediatric context, and two others were combined into one as they both concerned orthopaedic examinations (see attachment 2 ). These changes were made to clarify the EPAs and avoid misunderstandings. The students’ level of competency was self-assessed using the supervision scale from the PROFILES catalogue (see table 2 (Tab. 2)). Competency level 5 was not included as a response option, as medical students are not entrusted with this level of responsibility during their clerkships.

The questionnaire used in this study was in German. It was validated by a group of 3 recent medical graduates to ensure accurate understanding by the participants. It was distributed by the student affairs office of the medical faculty of the UZH via email and was verbally promoted during a lecture. All students who had completed a paediatric rotation were included in this study, regardless of the location of the teaching hospital they attended, in Zurich or abroad. Students who completed rotations only in paediatric surgery or paediatric psychiatry were not included in this study. An overall analysis was conducted initially without differentiating between the attended teaching hospitals. Subsequently, the different teaching hospitals were compared, if attended by a minimum of 10 students, to identify any significant differences in the teaching program, students’ experiences, or their achieved level of supervision in the EPAs.

Statistical analyses were performed using IBM^®^ SPSS^®^ statistics version 26 (IBM, Armonk, NY). Basic descriptive statistics and frequencies were used to describe all variables. The mean and standard deviation (SD) were used to describe the students’ assessment of the statements they were asked to rate, and boxplot analyses were used to calculate the median and depict the distribution of these answers. Descriptive statistics and bar diagrams were used to calculate and illustrate the distribution of students achieving respective levels.

In this study, an EPA was considered achieved if at least 2/3 of all students stated that they were able to perform the EPA at level 3 or higher.

## 3. Results

### 3.1. Demographic data

Out of 316 medical students attending their final year at the UZH, 189 responded to this survey, resulting in a response rate of 59.8%. 177 students attended a paediatric rotation at that year. Among the students who answered, 113 attended a paediatric rotation, 29 were trained at the University Children’s hospital Zurich, 22 at the children’s hospital in Luzern, 14 in Winterthur, 13 in St. Gallen and 10 in Bern. Further, paediatric clerkship was attended at 9 more children’s hospitals in Switzerland by 9 or less students per location while 3 students trained at children’s hospitals abroad (Ghana, Uganda and Kenia respectively). Most students worked at one hospital (93.7%) for a duration of 1-3 months (66.9%). The mean age of the participants was 25.6 years (SD 2.3), with 64.6% being female and 93.3% being native speakers of German.

### 3.2. Overall experience and satisfaction

The most common reason named for choosing a paediatric rotation during the WSJ was “interest in the paediatric discipline” (80.9%), and the most common expectation was “to gain insight” (93.6%). The overall experience was rated as “good” or “very good” by 77.1% of all students. In total, 87% of the students would recommend a paediatric rotation, with 71.4% of them recommending the teaching hospital they trained at. The rotation experience directly influenced the future career plans in 35.8% (39) of all student, leading hereof 56.5% (22) to pursue a paediatric residency, whilst 43.6% (17) left this track due to their experience during rotation regarding the everyday work and working condition of a paediatrician (see table 3 (Tab. 3)).

Special training modules were offered in 53.6% of paediatric clerkships, meanwhile 50.5 % of the students wished for more structured learning opportunities. However, these numbers varied greatly among individual teaching hospitals.

Regarding everyday work during rotation, students were mostly able to apply their knowledge and practice paediatric skills and were often given independent tasks and received teaching from medical educators. Overall, the feedback provided was mostly deemed helpful, and the students did often feel integrated into and valued by the clinical team. Regarding the Mini-CEX, students felt mostly neutral regarding the help it provided assessing their competencies and weaknesses and the feedback provided during these assessments. These results also varied among individual teaching hospitals, as differences in the curriculum and work culture were described, as well as differences regarding correct execution of the Mini-CEX. Each teaching hospital had advantages and drawbacks regarding at least one of these aspects when compared to the overall assessment. The student’s satisfaction further varied depending on which ward they were assigned to during their rotation and what kind of work they were asked to perform. A frequently noted comment in our survey included students deeming direct involvement in patient care and a certain level of independency working (with ensured timely backup by a resident or an attending) such as mostly possible in an emergency room a lot more educational than only doing paperwork while stationed at a ward. The logbook was not rated as useful in any way (see table 4 (Tab. 4)).

### 3.3. Level of competency reached in EPAs

The self-assessed level of competency achieved for different EPAs varied significantly. For 14 out of 26 EPAs, at least two-thirds of all students rated themselves at the expected level 3 or higher (indirect or distant supervision). A minimum of competency level 2 (close supervision) was acquired for a total of 23 EPAs.

For EPA 1 “Taking a medical history”, more than 2/3 of all students felt comfortable taking an age-specific paediatric history at level 3 or higher, hence it required more supervision when assessing the development and lifestyle of children or adolescents (acquired by 2/3 at a level 2 or higher).

For EPA 2 “Assess the physical and mental status of the patient”, the expected level of supervision (level 3 was acquired by at least 2/3) was met for the EPAs regarding general physical examination, interpretation of abnormal findings, and specific examinations of the cardiovascular, pulmonary, abdominal, head-and-neck, and neurological systems. However, orthopaedic examination of joints and spine, examination of the male genitals, assessment of age-specific body measurements, and assessment of age-specific development posed more difficulties and required closer supervision, as did the examination of newborns and pubertal growth, which less than 2/3 acquired level 2 or higher.

EPA 3 “Prioritize a differential diagnosis following a clinical encounter” and EPA 4 “Recommend and interpret diagnostic and screening tests in common situations” were not acquired at the expected level 3 by 2/3 of students. Closer supervision was needed to assess the degree of emergency, develop a differential diagnosis integrating scientific foundations, order and interpret imaging, and interpret test results and integrate them into the differential diagnosis (level 2 or higher was acquired by 2/3 of all students), as well as evidence-based, cost-effective ordering of tests (less than 2/3 acquired level 2 or higher). 

EPA 8 “Document and present patient’s clinical encounter; perform handover” was performed at level 3 by more than 2/3 of all students, as they were able to keep a patient’s chart and provide an organized oral presentation of a patient (see attachment 2 ).

**Figure 1:**
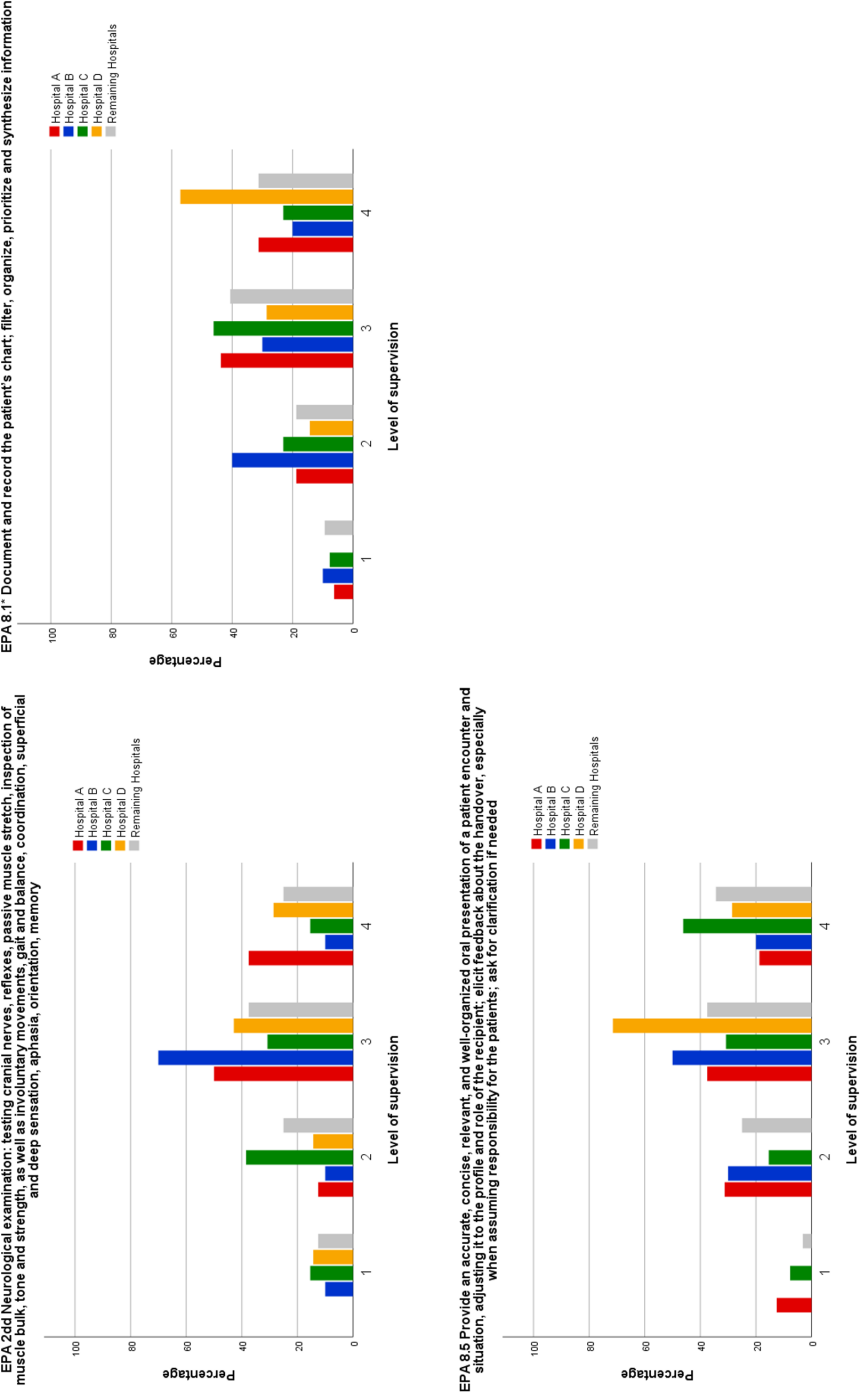
Comparison of teaching hospitals regarding EPAs which students acquired at a level 3 in the overall analysis but not in the individual analysis of each teaching hospital Note: Distribution of the level of supervision of Entrustable Professional Activities (EPAs) by teaching hospitals. These EPAs were assessed at a level 3 in the overall analysis. However, some students at certain teaching hospitals were unable to perform EPAs at the required level of supervision. Level of supervision is defined as (1) Students are only allowed to observe the EPA, (2) EPA can be performed under direct supervision, (3) EPA can be performed under indirect supervision, (4) EPA can be performed independently under distant supervision. Hospitals A – D represent four different teaching hospitals offering a paediatric rotation that were attended by at least 10 students. All remaining teaching hospitals offering a paediatric rotation that were attended by 9 or less students were summarized in one category.

**Figure 2:**
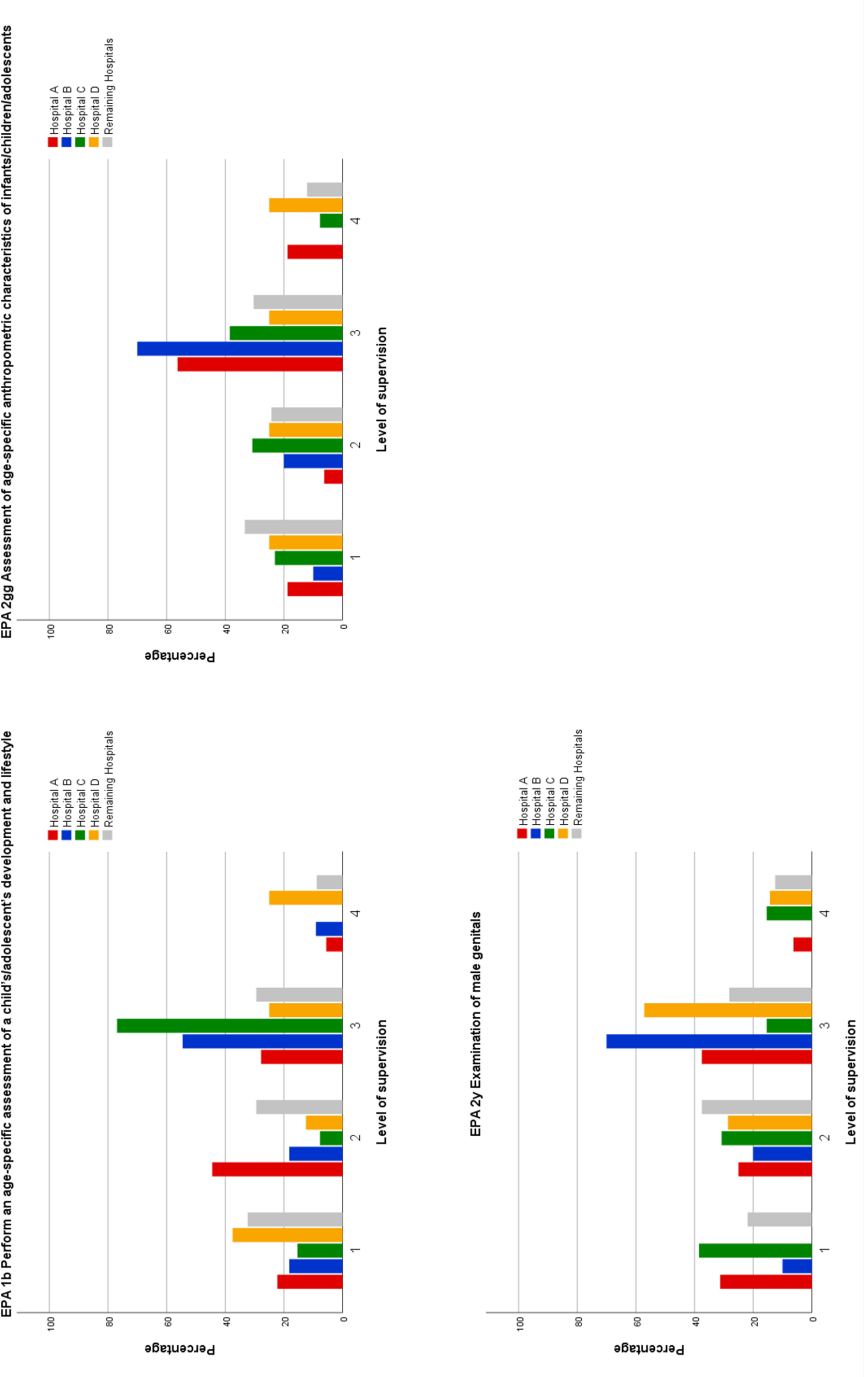
Comparison of teaching hospitals regarding EPAs which students did acquire at a level 3 in the individual analysis of some teaching hospitals but not in the overall analysis Note: Distribution of the level of supervision of Entrustable Professional Activities (EPAs) by teaching hospitals. These EPAs were assessed with a level 1 or 2 in the overall analysis. However, students at certain teaching hospitals did acquire the required level 3 of supervision. Level of supervision is defined as (1) Students are only allowed to observe the EPA, (2) EPA can be performed under direct supervision, (3) EPA can be performed under indirect supervision, (4) EPA can be performed independently under distant supervision. Hospital A – D represent four different teaching hospitals offering a paediatric rotation that were attended by at least 10 students. All remaining teaching hospitals offering a paediatric rotation that were attended by 9 or less students were summarized in one category.

Comparing the overall analysis to the individual results of the four teaching hospitals discrepancies were identified. Out of the 14 EPAs in which the expected indirect supervision was acquired in the overall analysis, results show that 3 EPAs were not attained at the required level by students of at least one teaching hospital (see figure 1 (Fig. 1)). Furthermore, out of the 12 EPAs that students were not able to perform at a level 3 in the overall analysis, results of the individual teaching hospital showed that students from at least one hospital did attain the demanded level of supervision in 3 EPAs (see figure 2 (Fig. 2)). 

Discrepancies between the overall and the individual analyses were identified for 6 EPAs, and for every teaching hospital, they were found for at least one EPA. However, these differences are likely more indicative of sample variance rather than a systemic effect due to sample size. 

## 4. Discussion

Clerkships in paediatrics, such as those offered during WSJ, provide an ideal opportunity for medical students to train clinical skills and competencies while gaining insight into the everyday clinical work of a paediatrician [10]. By the end of medical school education, students should have the competency to accurately conduct a thorough examination of a paediatric patient, formulate a differential diagnosis, and initiate appropriate examinations and therapy.

Our study revealed that overall, the paediatric clerkship as part of the curriculum at the medical school of the UZH was a positive experience for most students, with only 2.8% of students not recommending attending a paediatric rotation, despite 49.5% of them deciding not to pursue a paediatric residency. This underscores the value and importance that students attribute to this aspect of their education [11].

Discrepancies between the students’ perceptions of a successful rotation and the demands of the medical school curriculum were identified. While the PROFILES catalogue focuses on competencies and EPAs, students’ expectations are more diverse [12]. In our study, the majority of students (87.3%) expected to further improve their paediatric skills and knowledge during the rotation, but the most commonly cited expectation (93.6%) was the desire to gain insight into daily clinical routine – findings, that are consistent with current literature [11], [13], [14]. Goals such as the opportunity to gain hands-on experience, integration into the clinical team and routine, and the ability to receive appropriate supervision and competency-based feedback were paramount for them.

Currently, students were only able to perform 14 out of the 26 EPAs at the level of supervision expected by PROFILES at the end of medical school (self-reported). Most students felt comfortable performing medical history taking, general clinical examinations, and documentation of clinical encounters (EPAs 1, 2, and 8) without close supervision. These skills are regularly taught and practiced in various clinical situations during rotation and are included in students’ assessments [15]. However, students did not feel competent in performing specific tasks such as the examination of joints and spine, male genitals, examination of the newborn, and the assessment of age-specific development, body measurements and pubertal growth. These activities are encountered and practiced less frequently by students during their rotations. Orthopaedic examinations and assessments of age-specific development are also often directly referred to specialists when treatment is required. Additionally, for other EPAs, students did not have the opportunity to reach a level 3, as medical educators might be hesitant to allow distant supervision for activities such as the examination of newborns and male genitals, as well as the assessment of pubertal growth.

Students also expressed the need for closer supervision in prioritizing differential diagnoses and recommending and interpreting diagnostic examinations (EPAs 3 and 4). These activities are complex, as students are required not only to collect clinical data, but also to apply clinical reasoning for interpretation and decision making. During their rotations, students typically seek consultation from their supervisors prior to making such decision. As these skills are developed over time and through experience, it is not surprising that student have not yet acquired them at the expected level during their short rotations [15]. Our results are consistent with the study of Marty et al. [15] of former medical graduates from the UZH, who did not attain the expected level of supervision in a similar subset of EPAs, regardless of their specialty focus. It appears that this gap between expected and acquired competency levels is not unique to paediatrics. Furthermore, self-assessment of EPA competency levels should be interpreted with caution, as EPA assessment should also involve input from supervising personnel.

The cohort of students that was included in the study was one of the first to start their clinical training after the new national PROFILES framework was set up in 2018. The implementation of the new competency-based curriculum is in the hands of each medical school, the progress of implementation is ongoing. In the paper by Sohrmann et al. [2] the implementation of this new concept of EPAs nationwide in undergraduate medical education is described as an ongoing challenging process, that needs a collaborative approach to “develop synergies at the national level and to share the multiple implementation experiences”. Our study can add information about undergraduate training in paediatrics in the clinical environment and identify some gaps. To successfully implement the PORFILES catalogue into paediatric rotations, in our eyes adjustments to the current curriculum are necessary. Based on our findings, we propose measures to improve the level of competency achieved at the end of the rotation, which will enhance the overall positive student experience.

Students need to be more directly involved in patient care, taking on responsibilities and applying their knowledge and skills according to their capabilities [16], [17]. Acquiring, maintaining, and improving their level of supervision required in an EPA necessitates regular encounters with and practice of these skills [16], [18]. This is consistent with our findings that students perceive working at the emergency department as particularly educational, as they are capable to treat patients in non-life-threatening situations more independently, while experiencing frustration when stationed on a ward doing paperwork with less involvement in patient care.

As supervision and feedback are essential for competency training [19], medical educators should receive instruction and training on how to provide feedback and perform assessments, especially when assessing EPAs [9]. Individualized and qualitative feedback can improve students’ level of competency, helping them to understand their strengths and weaknesses [20], [21]. The entrustment decision of a superior is a prerequisite for progression to the next level in an EPA [16], [22]. Medical educators should actively provide supervision and feedback on a daily basis during rotations, in addition to structured assessments. It was further shown, that by implementing EPAs into clerkships, students can benefit from prompt and specific feedback [8]. Medical educators should as such be instructed to actively incorporate EPA training into everyday clinical work whenever possible, with a focus on EPAs that were not acquired at a level 3 but are rated as essential in paediatrics and relatively easy to learn and train, such as the use of percentiles to evaluate growth. Structured teaching courses can be offered for topics that students do not regularly encounter during rotations, an approach also chosen during the implementation of EPAs by other teaching hospitals [4], [8]. As such, we recommend implementing training modules into the WSJ for orthopaedic examination, examination of newborns, and interpretation of radiological imaging. Such training programs can help students to improve their level of competency and were highly appreciated according to our study [16]. Assessment tools such as the Mini-CEX can be used as a helpful tool to evaluate a student’s level of proficiency when performed correctly [23]. A Mini-CEX that includes structured, individualized feedback can improve the students’ experience as well as their level of competency [24]. This is consistent with our findings that students deemed the Mini-CEX only useful when done correctly. Therefore, it is essential that medical educators receive instructions on how to correctly perform these assessments. As students did not benefit from the logbook, alternatives should be discussed. Options may include technological solutions such as app-supported assessment tools for EPAs [8]. 

Another difficulty is that the EPAs as presented in the PROFILES catalogue are not specifically designed for paediatrics, and they often include skills and methods that are not commonly used in this field. Therefore, selection and adaptation of EPAs are necessary when designing a curriculum for paediatric rotations – a challenge also identified by other medical school when implementing EPAs into undergraduate medical education [8], [9]. Some EPAs may seem too ambitious for undergraduate training, especially if competency level 3 is expected, such as the EPAs related to the examination of a newborn, male genitals, assessment of a child’s development, as well as those involving differential diagnosis and ordering and interpretation of diagnostics. These EPAs may not be realistic goals for undergraduate training as students may not have the opportunity to reach distant supervision due to the high level of responsibility involved. In our opinion, reaching level 2, which is performing under direct supervision, in some EPAs is a more realistic and sufficient goal for undergraduate training (see table 2 (Tab. 2)). As the expected competency level at the end of undergraduate training marks the starting point of postgraduate residency training, an alignment with the paediatric postgraduate speciality curriculum should be aimed for.

Overall, the introduction of the PROFILES catalogue offers an opportunity to improve medical education and competency-based training in paediatric rotations. While students’ priorities go beyond just reaching competency levels in EPAs, these objectives constitute the foundation of a curriculum aimed at ensuring a hight educational quality by enhancing clinical skills and reasoning. With the implementation of the PROFILES catalogue, paediatric rotations will be able to achieve the goal of offering competency-based education. 

### 4.1. Limitations

Our survey was designed as a self-assessment of the level of supervision needed for the EPAs. However, entrustment decisions are usually made by clinical educators. They are based not only on knowledge and skills but also on attitude, such as the understanding of one’s limitations and the ability to recognize when help is needed, and are further influenced by the attributes and trust of the educator in a student’s capabilities [5], [7], [22], [25]. These aspects may not have been fully represented in our survey. Self-assessment can also result in over- or underestimation of actual abilities and skill levels [26]. Additionally, some EPAs include multiple examinations or skills in their description, which can lead to inaccuracies in self-assessment, as well as uncertainty about the actual meaning of the more complexly phrased EPAs (e. g. EPA 4.2 “Justify an informed, evidence-based rationale for ordering tests (when appropriate, based on integration of basic medical disciplines as they relate to the clinical condition); take into account cost-effectiveness of ordering”).

Another limitation is the sole use of a questionnaire to answer the research questions. A mixed-method approach with additional focus group discussions and interviews could have helped to deepen the understanding and interpretation of our qualitative results. Hence to get a broader picture it was rated more feasible to approach all students to fill out an electronic questionnaire, therefore reaching a high participation level. 

## 5. Conclusion

The majority of students reported satisfaction with their paediatric rotation during WSJ, with integration into the clinical team, receiving feedback and supervision from medical educators, and improving competency levels being important aspects. However, there is a need for better integration into clinical teams, increased supervision and feedback, and training of medical educations in paediatric clinical skills and competencies to further enhance the training experience. 

The implementation of EPAs in the clinical context aligns with these goals, although the role of EPAs and clinical skills assessments in paediatrics still needs to be defined. Some EPAs in the PROFILES catalogue may not be suitable for paediatrics, and there is a need to adapt certain EPAs or set a lower competency level as a more realistic goal for undergraduate medical education. 

### 5.1. Practice points

To improve the current education standard and the required level of supervision for EPAs, we suggest the implementation of the following measures in paediatric training:

- Instruction of faculty/medical teachers to actively provide supervision and feedback to students
- Students are entrusted to take over responsibility for patient care
- Implementation of teaching modules during rotation to train specific skills
- Adaptation of EPAs to paediatric specific skills, defining a level 2 supervision as sufficient for the EPAs regarding the examinations of newborns, male genitals, and the assessment of development

## Notes

### Authorship

The authors Michelle Seiler and Sabine Kroiss Benninger share the last authorship.

### Authors’ ORCIDs

- Lya Baumann: [0009-0002-8042-637X]
- Beatrice Latal: [0000-0003-1309-4790]
- Michelle Seiler: [0000-0002-1263-5818]
- Sabine Kroiss Benninger: [0000-0003-3009-1153]

## Competing interests

The authors declare that they have no competing interests. 

## Supplementary Material

Survey about the paediatric rotation during medical school

Entrustable Professional Activities by PROFILES, adjusted for our study

## Figures and Tables

**Table 1:**
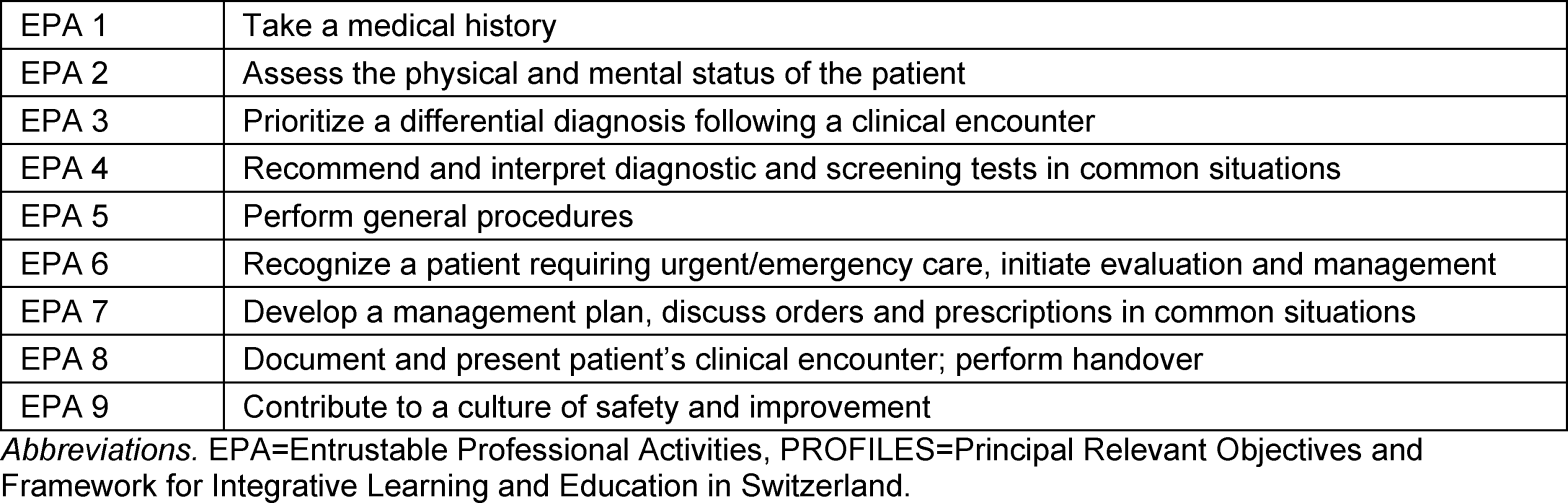
Core EPAs defined by PROFILES

**Table 2:**
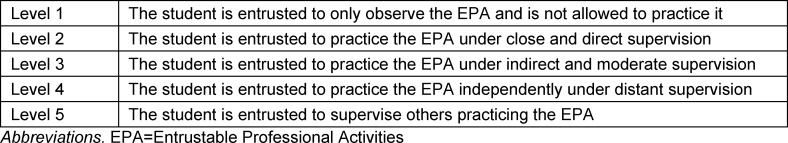
Entrustment and supervision scale for assessing EPAs

**Table 3:**
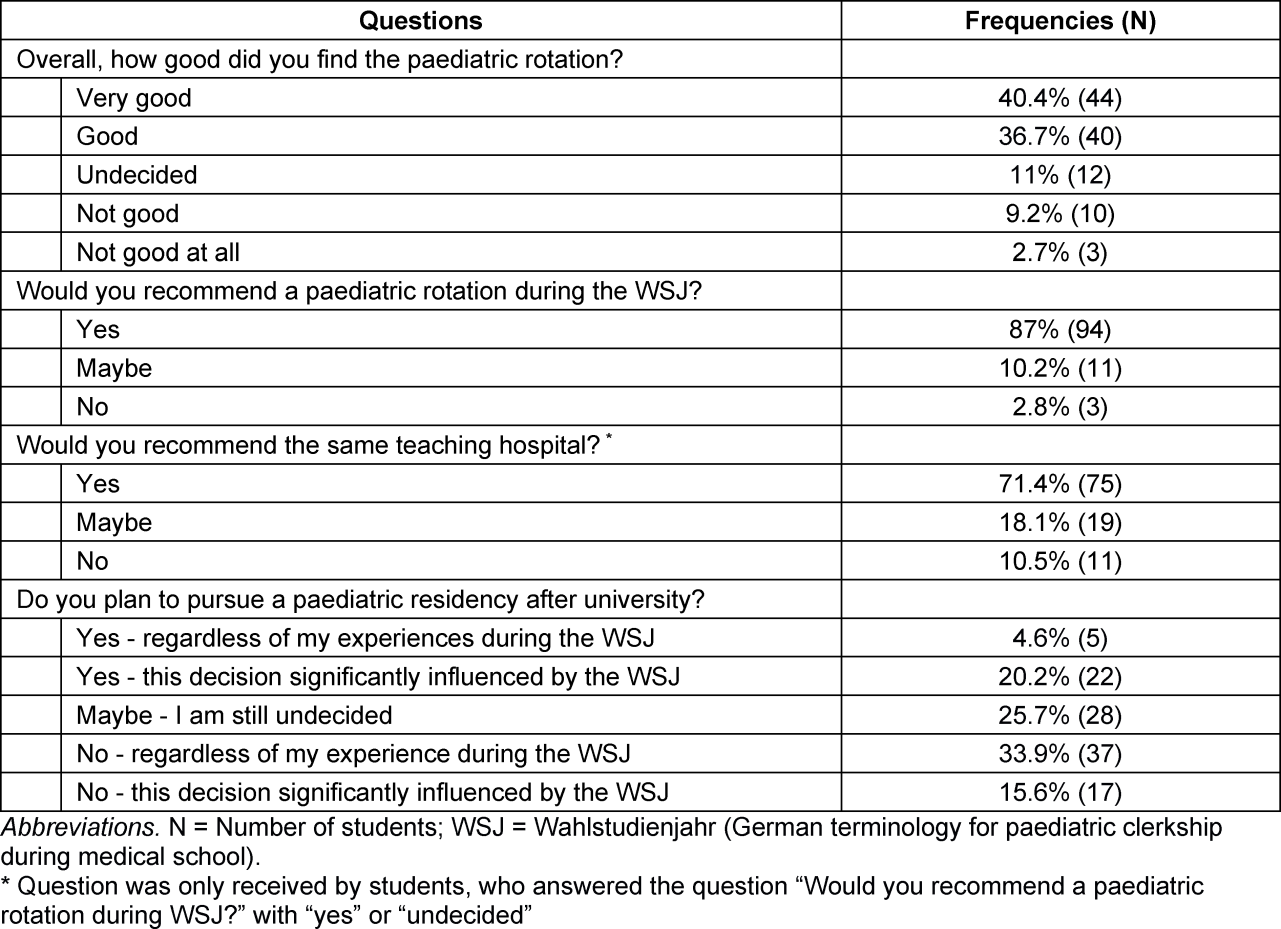
Overall satisfaction regarding the paediatric rotation during WSJ

**Table 4:**
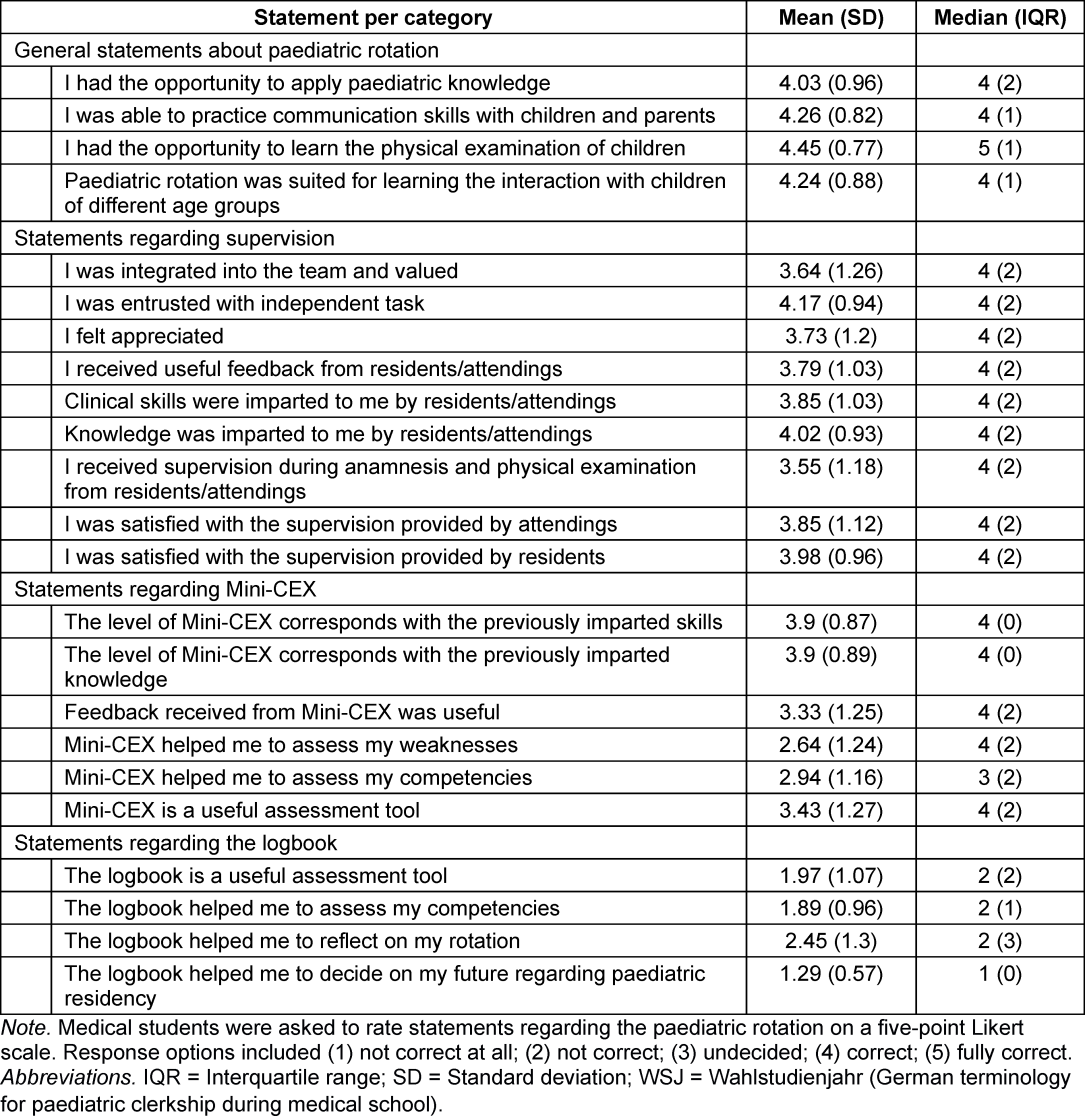
Statements regarding the paediatric rotation during WSJ
