# Supplementary material for: Paediatric rotations in undergraduate medical education in Switzerland: Meeting students’ expectations and the goals of the competency-based learning catalogue PROFILES
Source: GMS J Med Educ. 2024 Nov 15;41(5):Doc63. doi: 10.3205/zma001718 (PMC11656168; doi:10.3205/zma001718)
Supplement: Survey about the paediatric rotation during medical school [file JME-41-63-s-001.pdf]

Attachment 1: Survey about the paediatric rotation during medical school

Questionnaire for the dissertation on the reorganisation of the paediatric rotation

Thank you for participating in the survey regarding the expectations and experiences during paediatric rotation.

Participation is voluntary and all answers remain anonymous. If you have any questions, please contact us at: lya.baumann@uzh.ch

With kind regards

Lya Baumann, Doctoral student of human medicine, state exam 2019
Dr. med. Michelle Seiler, senior physician, paediatric emergency
Dr. med. Sabine Kroiss Benninger, senior physician mbF, paediatric oncology
Children's University hospital Zürich

Attachment 1 to Baumann L, Latal B, Seiler M, Kroiss Benninger S. *Paediatric rotations in undergraduate medical education in Switzerland: Meeting students' expectations and the goals of the competency-based learning catalogue PROFILES*. GMS J Med Educ. 2024;41(5):Doc63. DOI: 10.3205/zma001718

General information about the participant

- Age
  - 16-60

- Gender
  - Male
  - Female
  - Others

- Native language *(multiple answers possible)*
  - German
  - Italian
  - French
  - English
  - Others *(please specify)*:________________________

- Did you attend a paediatric rotation during the Wahlstudienjahr?
  - Yes
  - No
  - Only a rotation in paediatric surgery or paediatric psychiatry

- Why did you choose not to attend a paediatric rotation? *(multiple answers possible)* (Question only received if the prior question was answered with "No" or "Only a rotation paediatric surgery or paediatric psychiatry". The survey was completed with this question for all who received it.)

  - No interest in paediatrics
  - Was not able to get a place at a teaching hospital
  - Other reasons *(please specify)*:________________________

General questions regarding the paediatric rotation

- Where did you attend a paediatric rotation? *(multiple answers possible)*
  - Aarau, Kantonsspital Aarau
  - Bellinzona, Ospedale Regionale Bellinzona e Valli
  - Bern, Kinderkliniken Inselspital Bern
  - Basel, Universitäts-Kinderspital beider Basel
  - Chur, Kantonsspital Graubünden
  - Fribourg, Hôpital cantonal Fribourg
  - Lugano, Ospedale Regionale di Lugano
  - Luzern, LUKS Kinderspital
  - St. Gallen, Ostschweizer Kinderspital
  - Winterthur, Kantonsspital Winterthur
  - Zürich, Stadtspital Triemli
  - Zürich, Universitäts-Kinderspital Zürich
  - Others / At a paediatrician's practice *(please specify location)*:____________________

- What was the overall duration of your paediatric rotation?
  - ≤ 1 month
  - > 1 month – < 3 months
  - ≥ 3 months

- In which departments did you work? *(multiple answers possible)*
  - General paediatrics
  - Intensive care unit
  - Paediatric surgery
  - Neonatology
  - Emergency department
  - Others *(please specify)*:______________________

- What were your reasons to attend a paediatric rotation *(multiple answers possible)*
  - Interest in the paediatric speciality
  - Joy in working with children
  - Future career goals in paediatrics
  - Coincidence, no special reasons
  - Others *(please specify)*:______________________

- What were your expectations attending a paediatric rotation? *(multiple answers possible)*
  - Gaining insight into the paediatric specialty
  - Preparation for the final exam of medical school
  - Deepen paediatric knowledge and skills
  - Preparation for a future as a paediatrician
  - Others *(please specify)*:______________________

- Do you plan to pursue a paediatric residency after university?
  - Yes – I already planned that before and regardless of my experience during rotation
  - Yes – This decision was significantly influenced by my experience during rotation
  - Undecided – I may attend a year of paediatric residency
  - No – regardless of my experience during rotation I decided to pursue a residency in another speciality
  - No - this decision was significantly influenced by my experience during rotation

- Overall, how good did you find the paediatric rotation?
  - Not good at all
  - Not good
  - Undecided
  - Good
  - Very good

- Would you recommend a paediatric rotation during the Wahlstudienjahr?
  - Yes
  - Maybe
  - No

- Would you recommend a paediatric rotation at the same teaching hospital? (Question was only received if the students answered the prior question with "yes" or "maybe".)
  - Yes
  - Maybe
  - No

- Please rate the following general statements regarding the paediatric rotation on a five-point Likert scale.
  (1) not correct at all (2) not correct (3) undecided (4) correct (5) fully correct
  - Paediatric rotation was suited for learning the interaction with children of different age groups
  - I had the opportunity to learn the physical examination of children
  - I was able to practice communication skills with children and parents
  - I had the opportunity to apply paediatric knowledge

  - *Any Comments:*__________________________

- Rate the following statements regarding supervision and integration into the team during paediatric rotation on a five-point Likert scale.
  (1) not correct at all *(2) not correct* *(3) undecided* *(4) correct* *(5) fully correct*
  - I was satisfied with the supervision provided by residents
  - I was satisfied with the supervision provided by attendings
  - I received supervision during anamnesis and physical examinations from residents/attendings
  - Knowledge was imparted to me by residents/attendings
  - Clinical skills were imparted to me by residents/attendings
  - I received useful feedback from residents/attendings
  - I felt appreciated
  - I was entrusted with independent tasks
  - I was integrated into the team and valued

  - *Any Comments:*__________________________

- Were there any special training programs for medical students during rotation?
  - Yes
  - No

  - *If yes, please elaborate in what form:*__________________________

- Would you have wished for more structured training programs for medical students during rotation?
  - Yes
  - No

  - *If yes, please elaborate in what form:*__________________________

- Rate the following statements regarding the preparation for the paediatric rotation during university on a five-point Likert scale.
  (1) not correct at all *(2) not correct* *(3) undecided* *(4) correct* *(5) fully correct*
  - I felt well prepared for paediatric rotation
  - I would have wished for more paediatric bedside teaching
  - I would have wished for more knowledge to be imparted through lectures
  - I would have wished for more communication training
  - I would have liked more information about everyday clinical life in paediatrics

  - *Any Comments:*__________________________

- Rate the following statements regarding the Mini Clinical Evaluation Exercise (Mini-CEX) during paediatric rotation on a five-point Likert scale.
  (1) not correct at all (2) not correct (3) undecided (4) correct (5) fully correct
  - Mini-CEX is a useful assessment tool
  - It helped me to assess my competencies in paediatrics
  - It helped me to assess my weaknesses in paediatrics
  - Feedback received from Mini-CEX was useful
  - The level of Mini-CEX corresponds with the previously imparted knowledge
  - The level of Mini-CEX corresponds with the previously imparted skills

  - *Any Comments:*__________________________

- Rate the following statements regarding the logbook ("Logbuch") during paediatric rotation on a five-point Likert scale.
  (1) not correct at all (2) not correct (3) undecided (4) correct (5) fully correct
  - I find the Logbuch to be useful
  - It helped me to assess my competencies
  - It helped me to reflect on the rotation
  - It helped me with my decision regarding a potential future in paediatrics

  - *Any Comments:*__________________________

Level of competency in Entrustable Professional Activities (EPAs)

The Swiss PROFILES catalogue lists the learning objectives for medical students as Entrustable Professional Activities (EPAs). These medical activities of everyday clinical practice and their correct and safe execution are learned during medical school and postgraduate training. The level of competence achieved in an EPA can be measured by the degree of supervision required from a medical educator while performing the activity.

Please assess your level of competency in the following EPAs as attained at the end of your rotation:

1 = The student is entrusted to only observe the EPA and is not allowed to practice it
2 = The student is entrusted to practice the EPA under close and direct supervision, the medical educator is at all times in the same room, observing
3 = The student is entrusted to practice the EPA under indirect and moderate supervision, the medical educator is nearby and immediately on site in case of uncertainty, but must actively be involved
4 = The student is entrusted to practice the EPA independently under distant supervision, the instructor can be reached by telephone or will be on site within 30 minutes

Please focus specifically on paediatrics while rating your competency in those EPAs.

EPA 1. Medical history

- 1a Take an age-specific paediatric history (involving mother/father and child or adolescent)

  (1) only observing *(2) close supervision* *(3) indirect supervision* *(4) independently*

- 1b Perform an age-specific assessment of a child’s / adolescent’s development and lifestyle

  (1) only observing *(2) close supervision* *(3) indirect supervision* *(4) independently*

EPA 2. General clinical examination of a child

- 2.1 Perform an accurate and clinically relevant physical examination in a logical and fluid sequence, with a focus on the purpose and the patient’s expectations, complaints and symptoms, in persons of all ages

  (1) only observing *(2) close supervision* *(3) indirect supervision* *(4) independently*

Attachment 1 to Baumann L, Latal B, Seiler M, Kroiss Benninger S. *Paediatric rotations in undergraduate medical education in Switzerland: Meeting students’ expectations and the goals of the competency-based learning catalogue PROFILES*. GMS J Med Educ. 2024;41(5):Doc63. DOI: 10.3205/zma001718

- 2.4 Identify, describe, document and interpret abnormal findings of a physical examination. Assess vital signs (temperature, heart and respiratory rate, blood pressure)

  (1) only observing (2) close supervision (3) indirect supervision (4) independently

- 2.5 Demonstrate patient-centred examination techniques; demonstrate effective use of devices such as a stethoscope, otoscope, ophthalmoscope; respect patient privacy, comfort, and safety

  (1) only observing (2) close supervision (3) indirect supervision (4) independently

- 2a Assessment of patient's general condition and vital signs

  (1) only observing (2) close supervision (3) indirect supervision (4) independently

- 2b Assessment of nutritional status

  (1) only observing (2) close supervision (3) indirect supervision (4) independently

- 2gg Assessment of age-specific anthropometric characteristics of infants / children / adolescents

  (1) only observing (2) close supervision (3) indirect supervision (4) independently

- 2hh Assessment of pubertal growth (pubertal stages)

  (1) only observing (2) close supervision (3) indirect supervision (4) independently

- 2ii Age-specific assessment of the child: neurological and cognitive development

  (1) only observing (2) close supervision (3) indirect supervision (4) independently

EPA 2. Specific clinical examination of a child

- 2k* (EPAs which were adjusted for our survey and did not correspond with the original EPA as published by the PROFILES catalogue were labelled with a star (*).) Inspection and palpation of auricle and adjacent region as well as external auditory canal and tympanic membrane

  (1) only observing (2) close supervision (3) indirect supervision (4) independently

- 2p/2q* Orthopaedic Status: Functional testing of joint mobility: (shoulders, elbows, wrists, fingers, hips, knees and ankles); Inspection, palpation, percussion and mobility of the spine

  (1) only observing (2) close supervision (3) indirect supervision (4) independently

- 2r Inspection and palpation of chest, percussion and auscultation of lungs

  (1) only observing (2) close supervision (3) indirect supervision (4) independently

- 2s Palpation (apex beat/fremitus) and auscultation of heart; description of normal/abnormal heartbeat and murmurs

  (1) only observing (2) close supervision (3) indirect supervision (4) independently

- 2t* Palpation of pulse

  (1) only observing (2) close supervision (3) indirect supervision (4) independently

- 2w Palpation, percussion and auscultation of abdomen, description of findings

  (1) only observing (2) close supervision (3) indirect supervision (4) independently

- 2y Examination of male genitals

  (1) only observing (2) close supervision (3) indirect supervision (4) independently

- 2dd Neurological examination: testing cranial nerves, reflexes, passive muscle stretch, inspection of muscle bulk, tone and strength, as well as involuntary movements, gait and balance, coordination, superficial and deep sensation, aphasia, orientation, memory

  (1) only observing (2) close supervision (3) indirect supervision (4) independently

- 2ff Examination of new-borns (Apgar score, dysmorphism, malformation)

  (1) only observing (2) close supervision (3) indirect supervision (4) independently

Attachment 1 to Baumann L, Latal B, Seiler M, Kroiss Benninger S. *Paediatric rotations in undergraduate medical education in Switzerland: Meeting students' expectations and the goals of the competency-based learning catalogue PROFILES*. GMS J Med Educ. 2024;41(5):Doc63. DOI: 10.3205/zma001718

EPA 3. Assessment and differential diagnosis

- 3.2 Assess the degree of urgency of any complaint, symptom or situation

  (1) only observing (2) close supervision (3) indirect supervision (4) independently

- 3.4 Integrate the scientific foundations of basic medical sciences as well as epidemiological information (probability of diseases) into clinical reasoning, in order to develop a differential diagnosis and a working diagnosis, organized in a meaningful hierarchical way

  (1) only observing (2) close supervision (3) indirect supervision (4) independently

EPA 4. Diagnostics

- 4.2 Justify an informed, evidence-based rationale for ordering tests (when appropriate, based on integration of basic medical disciplines as they relate to the clinical condition); take into account cost-effectiveness of ordering

  (1) only observing (2) close supervision (3) indirect supervision (4) independently

- 4.5 Interpret test results and integrate them into the differential diagnosis; understand the implications and urgency of an abnormal result and seek assistance with interpretation if needed

  (1) only observing (2) close supervision (3) indirect supervision (4) independently

- 4.7 Provide an informed rationale for ordering imaging examinations; interpret first-line, common X-rays; integrate diagnostic imaging into the clinical workup

  (1) only observing (2) close supervision (3) indirect supervision (4) independently

EPA 8. Medical history

- 8.1* Document and record the patient's chart; filter, organize, prioritize and synthesize information

  (1) only observing (2) close supervision (3) indirect supervision (4) independently

- 8.5 Provide an accurate, concise, relevant, and well-organized oral presentation of a patient encounter and situation, adjusting it to the profile and role of the recipient; elicit feedback about the handover, especially when assuming responsibility for the patients; ask for clarification if needed

  (1) only observing (2) close supervision (3) indirect supervision (4) independently

Conclusion (Answers optional)

- What do you think was particularly valuable and well done during paediatric rotation?

  ____

- Which of your expectations regarding the paediatric rotation were not met, what was bad or disappointing?

  ____

- Are there any concrete wishes or suggestions to improve the paediatric rotation that you would like to make?

  ____

- Final comments:

  ____

- Voluntary disclosure of your e-mail address for further enquiries if needed:

  ____

Thank you very much for your participation!!

Attachment 1 to Baumann L, Latal B, Seiler M, Kroiss Benninger S. *Paediatric rotations in undergraduate medical education in Switzerland: Meeting students' expectations and the goals of the competency-based learning catalogue PROFILES*. GMS J Med Educ. 2024;41(5):Doc63. DOI: 10.3205/zma001718
