# Supplementary material for: Paediatric rotations in undergraduate medical education in Switzerland: Meeting students’ expectations and the goals of the competency-based learning catalogue PROFILES
Source: GMS J Med Educ. 2024 Nov 15;41(5):Doc63. doi: 10.3205/zma001718 (PMC11656168; doi:10.3205/zma001718)
Supplement: Entrustable Professional Activities by PROFILES, adjusted for our study [file JME-41-63-s-002.pdf]

Attachment 2: Entrustable Professional Activities by PROFILES, adjusted for our study

		Entrustable Professional Activities	Percentage of students attaining level 3 or more	Percentage of students attaining level 2 or more
EPA 1. Take a medical history				
	1a	Take an age-specific paediatric history (involving mother/father and child or adolescent)	96.4%	98.8%
	1b	Perform an age-specific assessment of a child's/adolescent's development and lifestyle	47.6%	73.8%
EPA 2. Assess the physical and mental status of the patient				
	2.1	Perform an accurate and clinically relevant physical examination in a logical and fluid sequence, with a focus on the purpose and the patient's expectations, complaints and symptoms, in persons of all ages	93.8%	98.8%
	2.4	Identify, describe, document and interpret abnormal findings of a physical examination. Assess vital signs (temperature, heart and respiratory rate, blood pressure)	81.3%	98.8%
	2.5	Demonstrate patient-centred examination techniques; demonstrate effective use of devices such as a stethoscope, otoscope, ophthalmoscope; respect patient privacy, comfort, and safety	93.8%	97.5%
	2a.	Assessment of patient's general condition and vital signs	87.5%	95.0%
	2b.	Assessment of nutritional status	80.0%	90.0%
	2k.*	Inspection and palpation of auricle and adjacent region as well as external auditory canal and tympanic membrane	88.5%	97.4%
	2p./2q.	*Orthopaedic Status*: Functional testing of joint mobility: *(*shoulders, elbows, wrists, fingers, hips, knees and ankles*)*; Inspection, palpation, percussion and mobility of the spine	51.3%	83.3%
	2r.	Inspection and palpation of chest, percussion and auscultation of lungs	93.6%	98.7%
	2s.	Palpation (apex beat/fremitus) and auscultation of heart; description of normal/abnormal heartbeat and murmurs	83.3%	98.7%
	2t.*	Palpation of pulse	89.7%	97.4%
	2w.	Palpation, percussion and auscultation of abdomen, description of findings	96.2%	98.7%
	2y.	Examination of male genitals	46.2%	76.9%
	2dd.	Neurological examination: testing cranial nerves, reflexes, passive muscle stretch, inspection of muscle bulk, tone and strength, as well as involuntary movements, gait and balance, coordination, superficial and deep sensation, aphasia, orientation, memory	67.9%	89.7%
	2ff.	Examination of newborns (Apgar score, dysmorphism, malformation)	23.1%	65.4%
	2gg.	Assessment of age-specific anthropometric characteristics of infants/children/adolescents	53.8%	75.0%
	2hh.	Assessment of pubertal growth (pubertal stages)	18.8%	47.5%
	2ii.	Age-specific assessment of the child: neurological and cognitive development	23.8%	70.0%

Entrustable Professional Activities			Percentage of students attaining level 3 or more	Percentage of students attaining level 2 or more
EPA 3. Prioritize a differential diagnosis following a clinical encounter				
	3.2	Assess the degree of urgency of any complaint, symptom or situation	48.7%	88.5%
	3.4	Integrate the scientific foundations of basic medical sciences as well as epidemiological information (probability of diseases) into clinical reasoning, in order to develop a differential diagnosis and a working diagnosis, organized in a meaningful hierarchical way	44.9%	87.2%
EPA 4. Recommend and interpret diagnostic and screening tests in common situations				
	4.2	Justify an informed, evidence-based rationale for ordering tests (when appropriate, based on integration of basic medical disciplines as they relate to the clinical condition); take into account cost-effectiveness of ordering	21.8%	65.4%
	4.5	Interpret test results and integrate them into the differential diagnosis; understand the implications and urgency of an abnormal result and seek assistance with interpretation if needed	39.7%	82.1%
	4.7	Provide an informed rationale for ordering imaging examinations; interpret first-line, common X-rays; integrate diagnostic imaging into the clinical workup	29.5%	83.3%
EPA 8. Document and present patient's clinical encounter; perform handover				
	8.1*	Document and record the patient's chart; filter, organize, prioritize and synthesize information	70.5%	92.3%
	8.5	Provide an accurate, concise, relevant, and well-organized oral presentation of a patient encounter and situation, adjusting it to the profile and role of the recipient; elicit feedback about the handover, especially when assuming responsibility for the patients; ask for clarification if needed	71.8%	94.9%
Total number of EPAs in which at least 66.66% of the students attained the respective levels			*14*	*23*

Note. Medical students were asked to assess their level of competency regarding each of these EPAs at the end of the paediatric rotation, using the required level of supervision as a scale. Response options included: Level 1=Students are only allowed to observe the EPA; Level 2=EPA can be performed under direct supervision; Level 3=EPA can be performed under indirect supervision, Level 4=EPA can be performed independently under distant supervision.

Abbreviation. EPA=Entrustable Professional Activities; PROFILES=Principal Relevant Objectives and a Framework for Integrative Learning and Education in Switzerland.

* EPAs were adjusted for our study by deleting the crossed-out sentences/words of the original EPA by PROFILES; they were marked accordingly in the survey. EPA2p/2q was subsumed for our study into one EPA regarding orthopaedic examination, adjusting the original phrasing by adding the words in italic
